# The utilization and barriers of Pap smear among women with visual impairment

**DOI:** 10.1186/s12939-016-0354-4

**Published:** 2016-04-12

**Authors:** Wen-Hui Fang, Chia-Feng Yen, Jung Hu, Jin-Ding Lin, Ching-Hui Loh

**Affiliations:** Department of Family and Community Medicine, Tri-Service General Hospital, National Defense Medical Center, Taipei, Taiwan; Department of Public Health, Tzu-Chi University, Hualien, Taiwan; Medical Quality Department, E-DA Hospital, Kaohsiung, Taiwan; School of Public Health, National Defense Medical Center, Taipei, Taiwan

**Keywords:** Pap smear screening, Women with visual impairment, Utilization, Barriers

## Abstract

**Background:**

Many evidences illustrate that the Pap smear screening successfully reduces if the cervical cancer could be detected and treated sufficiently early. People with disability were higher comorbidity prevalence, and less likely to use preventive health care and health promotion activities. There were also to demonstrate that people with visual impairment has less access to appropriate healthcare services and is less likely to receive screening examinations. In Taiwan, there was no study to explore utilization of Pap smear, associated factors and use barriers about Pap smear screening test among women with visual impairment.

The purpose is to explore the utilization and barriers of using Pap smear for women with visual impairment in Taiwan. To identify the barriers of women with visual from process of receiving Pap smear screening test.

**Methods:**

The cross-sectional study was conducted and the totally 316 participators were selected by stratified proportional and random sampling from 15 to 64 year old women with visual impairment who lived in Taipei County during December 2009 to January 2010. The data was been collected by phone interview and the interviewers were well trained before interview.

**Results:**

The mean age was 47.1 years old and the highest percentage of disabled severity was mile (40.2 %). Totally, 66.5 % of participators were ever using Pap smear and 38.9 % used it during pass 1 year. Their first time to accept Pap smear was 38.8 year old. There was near 50 % of them not to be explained by professionals before accepting the Pap smear. For non-using cases, the top two percentage of barriers were “feel still younger” (22.3 %), the second was “there’s no sexual experience” (21.4 %). We found the gynecology experiences was key factor for women with visual impairment to use Pap smear, especially the experiences was during 1 year (OR = 4).

**Conclusions:**

Associated factors and barriers to receive Pap smear screening test for women with visual impairment can be addressed through interventions aimed at improving on cognitions and attitudes for cervical cancer risk factors. Our study would be as a reference resource for erasing the barriers and inequality among the visually disabled women.

## Background

Cervical cancer is the 3rd most common cancer in women around the world [10]. In Taiwan, cervical cancer is also the major cause of female cancer death. Fortunately, Pap smear, the useful screening tool, let the prevention and treatment of cervical cancer stride forward. Through the use of Pap smear screening, we can detect the precancerous lesions furthermore cancerous lesions at an earlier stage [35]. Many evidences illustrate that the Pap smear screening successfully reduces if the cervical cancer could be detected and treated sufficiently early, the cure rate of cancer can be as high as 70 to 90 % [1, 2, 7, 37]. The Pap smear screening test is current the most widely used approach for preventing cervical cancer with three to five yearly screening depending on the environment [30]. The benefit of Pap smear screening test in preventing cervical cancer has been demonstrated by in countries like United States and Taiwan. Since the use of Pap smear screening test in United States in the mid-20th century, cervical cancer, once the most frequent cause of death in women, now ranks for 14th cancer death. In Taiwan, the incidence rate of cervical cancer from 50.5 per 100,000 in 1995 drops to 21.1 per 100,000 in 2010 as well as the standardized mortality rate of cervical cancer also from 22.0 per 100,000 in 1995 falls to 8.2 per 100,000 in 2011 after the National Pap smear screening test program providing since 1995. The policy of Pap smear in Taiwan is that women who aged 30- year-old or more should receive once Pap smear screen in 3 years. This heathy service is free and its financial supports by HPA [12].

People with disability were higher comorbidity prevalence and medical utilization [22–25, 45], and less likely to use preventive health care and health promotion activities [19, 22, 41, 43, 44]. There were also to demonstrate that people with visual impairment has less access to appropriate healthcare services and is less likely to receive screening examinations (Hsu et al., 2009), as with individuals with other disable populations [5, 9]. Some of them showed the utilization of Pap smear in women with disability is significantly lower than without [3, 16]. Armour, et al. [3] found women with a disability were less likely than those without a disability to report receiving a Pap smear screening test during the past 3 years (78.9 % vs. 83.4 %; *p* < .001) in the 2008 Behavior Risk Factor Surveillance System in United States. Although the Pap smear utilization rate is much lower among disable population, they are usually in health disparity in most studies.

In Taiwan, there was no study to explore utilization of Pap smear, associated factors and use barriers about Pap smear screening test among women with visual impairment. The purposes of the present study are to explore the utilization and barriers of using Pap smear for women with visual impairment in Taiwan. To identify the barriers of women with visual from process of receiving Pap smear screening test can supply the references of primary health care strategies to reduce the disparity for them.

## Methods

This was a cross-sectional study and the population was 15–64 year old women with visual impairment who were officially registered in the Taiwan Disability Eligibility Determination System in Taipei County which was the most populous with vision disability city of Taiwan during December 2009 to January 2010. This study conducted for the government - Bureau of Health Promotion, Department of Health (the ID of plan: 9805006A) which aimed to evaluate the health policy effectiveness for the study group 2009. According to the IRB protocol in our institution at that time, this study was exempt from review which contacted by the government authority to the policy evaluation. The sampling design was stratified proportional and random sampling (stratified random sampling). The data was been collected by phone interview during 2009 and the interviewers were well trained in professional training courses before interview. When we start to interviews, we explained all the purposes of this study and related matters, and provide our contact way to samples for answer their doubts. During interviewed, they can go back at any time and reject the interview, we must respect their will. The oral informed consent was obtained while initial interview in eligible participant or her legally authorized representatives, which adhered to the guidelines of Declaration of Helsinki.

### Participates

When sampling error was ±0.05, the representative samples were estimated at the least 308 and then the stratified proportional and random sampling based on age to be employed in this study. We consider the valid response rate is 50 %, therefore, we oversampling as 616 participants. Finally, this study valid response samples was 316 who were 15–64 year old women with visual impairment in Taipei Country [46]. To compare with the population characteristics, there were no significant differences in age and seriousness of disability between our samples and population (*p* > 0.05) [46].

### The instrument and data collection

The instrument of our study was structured questionnaire which included the demographical characteristics, history of Pap smear test, and the experiences of barriers for Pap smear using that internal reliability were between 0.72 and 0.92 (Cronbach’s alpha). The experiences of barriers were parted into the negative attitudes of main care giver and environmental barriers. The data was been collected by three trained interviewers who were proficient in Mandarin and Hokkien (Taiwan dialect) and phone called at AM 10:00- PM 9:00. If the actual effective sample size was less than the expected estimation, we filled vacancies according to the random sampling rule again until a successful interview.

### Data analysis

The data were analyzed using the Statistical Package for the Social Sciences (vers. 20.0, SPSS, Chicago, IL, USA). The differences between the groups were considered significant if *P* values were 0.05.

Using a Pearson Chi-square test and a Fisher’s exact test, we compared differences in age, marriage, educational level, working status, severity of disability, and the causes of disability between no experience and ever using Pap smear for cases. We also used the logistic regression to explore the relational factors of using Pap smear for women with visual impairment.

## Result

We analyzed 316 women with visual impairment who were women with disability in eligibility determination system of Taiwan. Totally, 66.5 % of participators were ever using Pap smear in this study and 38.9 % used it during pass 1 year.

### Demographic data and factors of Pap smear using experience

The mean age was 47.1 years old and the highest percentage of disabled severity was mile (40.2 %). For our participators with using Pap smear, the mean age of the first time to accept Pap smear was 38.8 year old. Table 1 show the results of characters and comparing the differences between never and ever using Pap smear, there were significant different in age, marriage status, educational level, working status, the disabled causes, gynecology experiences and main care giver between two groups (*p* < 0.05). There were non-significant different in “Prefer gynecologist with female” and “Did you get any suggestions about pap smear from caregiver”.

**Table 1:** The Pap smear utilization by demographic characteristics among women with visual impairments, with *t*-test and Chi-square significance indicated

Variables	No experience *n* = 106 (%)	Ever using *n* = 210 (%)	Chi-square/*t*-test
Demographic characteristics			
Age (*n* = 316)	41.44 ± 15.60	49.95 ± 8.85	−5.210***
15 ~ 29	36 (34.0)	6 (2.9)	59.139***
30 ~ 64	70 (66.0)	204 (97.1)	
Marriage (*n* = 306)			
Married	50 (48.1)	171 (84.7)	75.701***
Unmarried	50 (48.1)	12 (5.9)	
Others	4 (3.8)	19 (9.4)	
Education (*n* = 292)			
Elementary school diploma and lower	22 (22.4)	77 (39.7)	15.913***
High school diploma	54 (34.8)	101 (52.1)	
University and higher	22 (55.2)	16 (8.2)	
Employee status (*n* = 306)			
No	66 (63.5)	174 (86.1)	33.857***
Yes	25 (24.0)	28 (13.9)	
Student	13 (12.5)	0 (0.0)	
Severity of disability (*n* = 316)			
Mild	32 (30.2)	95 (45.2)	6.641*
Moderate	37 (34.9)	57 (27.1)	
Severe	37 (34.9)	58 (27.7)	
The causes of disability (*n* = 292)			
Inborn (congenital)	41 (39.8)	49 (25.9)	101.93***
Acquired	62 (60.2)	140 (74.1)	
Gynecology experiences (*n* = 313)			
No	43 (41.0)	3 (1.4)	101.93***
Ever: before 1 year	39 (37.1)	66 (31.7)	
Ever: during 1 year	23 (21.9)	139 (66.9)	
Prefer gynecologist with female (*n* = 306)			
No	38 (37.3)	93 (45.6)	1.929
Yes	64 (62.7)	111 (54.4)	
Main care giver (*n* = 312)			
Care-self	6 (5.8)	6 (2.9)	74.845***
Mother and sisters	49 (47.1)	14 (6.7)	
Daughter and daughter in law	26 (25.0)	91 (43.8)	
Husband	19 (18.3)	81 (38.9)	
Others	4 (3.8)	16 (7.7)	
Did you get any suggestions about pap smear from caregiver (*n* = 275)			
No	67 (80.7)	149 (77.6)	0.334
Yes	16 (19.3)	43 (22.4)	

*p < 0.05, ***p < 0.001

### The cognitions, attitudes and barriers of Pap smear utilization among women with visual impairments

**Fig. 1:**
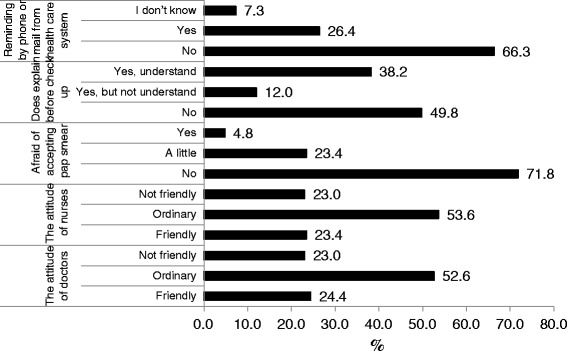
The barriers of using Pap smear for women with visual impairment (*n* = 202)

**Fig. 2:**
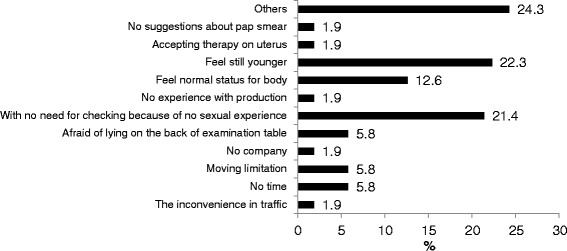
The causes of non-using Pap smear for people with visual impairment (*n* = 104)

Table 2 shows that there were non-significant different in recognition and the sufficient information of Pap smear (*p* > 0.05). There was near 50 % cases who ever used the Pap smear not to be explained that by professionals before they accepting the Pap smear. Figure 1 shows the barriers of Pap smear using, there were near 50 % of them to express that there were no explanation about Pap smear before checking up and the attitudes from doctors and nurses were more important than other factors. Figure 2 is to explain why they didn’t accept Pap smear, the highest percentage was “feel still younger” (22.3 %), the second was “there’s no sexual experience” (21.4 %).

**Table 2:** The cognitions, attitudes and barriers of Pap smear utilization among women with visual impairments

Variables	No experience *n* = 106 (%)	Ever using *n* = 210 (%)	Chi-square/*t*-test
Do you have any barriers for medical utilization (*n* = 291)			
No	85 (85.9)	160 (83.3)	0.313
Yes	14 (14.1)	32 (16.7)	
Do you know the “Pap smear” (*n* = 316)			
No	3 (2.8)	0 (0.00)	3.160***
Yes			
Never using	103 (97.2)	0 (0.00)	
Have used	0 (0.00)	210 (100.0)	
Sufficient information of pap smear (*n* = 308)			
Insufficient	52 (50.5)	126 (61.5)	5.157
Ordinary	36 (35.0)	47 (22.9)	
Not insufficient	15 (14.5)	32 (15.6)	
Know HPV vaccination (*n* = 307)			
Yes	52 (49.5)	119 (58.9)	2.467
No	53 (50.5)	83 (41.1)	
HPV is the major cause of cervical cancer (*n* = 306)			
Yes	64 (61.5)	119 (58.9)	0.197
No	40 (38.5)	83 (41.1)	
Still using pap smear regularly if accept HPV vaccination (*n* = 305)			
Yes	25 (24.0)	48 (23.9)	0.001***
No	79 (76.0)	153 (76.1)	
The mean age of the first time to accept pap smear	*NT*	38.8 ± 10.6	

*NT* no testing

### The associated factors of pap- smear utilization among women with visual impairment

Table 3 is the result of promoting factors (association) for using Pap smear. After controlling the age, marriage, educational level, employee status, severity of disability, the causes of disability, and the relationship of main care giver in the logical regression, we found the gynecology experiences was key factor for women with visual impairment to use Pap smear, especially the experiences was during 1 year (OR = 4, *p* < 0.001; adjust *R*^2^ = 55.2 %).

**Table 3:** The effect factors of pap smear utilization among women with visual impairment: Logistic regression^a^

Various	B	Exp (B)	*p*-value	95 % CI
Constant	−1.468	0.230		
Age	−0.041	0.960	0.100	0.914–1.008
Marriage				
Married	reference			
Unmarried	−0.578	0.561	0.486	0.110–2.856
Others	1.177	3.243	0.190	0.558–18.865
Education				
Elementary school diploma and lower	0.833	2.300	0.212	0.622–8.502
High school diploma	0.411	1.509	0.497	0.460–4.951
University and higher	reference			
Employee status				
No	reference			
Yes	0.030	1.031	0.954	0.372–2.858
Student	−18.266	<0.001	0.999	–
Severity of disability				
Mild	reference			
Moderate	−0.587	0.556	0.182	0.235–1.316
Severe	−0.802	0.449	0.059	0.195–1.030
The causes of disability				
Inborn (congenital)	reference			
Acquired	0.079	1.082	0.849	0.480–2.441
Gynecology experiences				
No	reference			
Ever: before 1 year	2.127	8.393^**^	0.002	2.131–33.048
Ever: during 1 year	3.996	54.403^***^	<0.001	13.236–223.609
Main care giver				
Care-self	reference			
Mother and sisters	−0.392	0.675	0.672	0.110–4.150
Daughter and daughter in law	1.876	6.530	0.079	0.802–53.130
Husband	2.050	7.765	0.058	0.935–64.467
Others	1.723	5.601	0.124	0.625–50.178
	R^2^CS = 0.400, R^2^N = 0.552			

^a^The reference group is no experience in using Pap smear

**p < 0.01, ***p < 0.001

### The barriers of using Pap smear for women with visual impairment

Among having experiences for Pap smear using group in this study, the barriers were from doctors and nurses attitudes about a quarter (25 %) and there was about 50 % of them thinking that there was no explain the procedure or information about Pap smear before screening. About 66.3 % of them didn’t get any reminding of re-check by mail or phone call from health care system. But over 70 % didn’t afraid of accepting Pap smear (Fig. 1).

Besides the cause of “others”, the top three proportions of the causes of never using Pap smear group were “feel still younger (22.3 %)”, “there is no sexual experience so don’t need to do (21.4 %)” and “feel healthy so don’t need to do (12.6 %)”.

## Discussion

The present study was the first survey of behavior and barriers for Pap smear using among women with vision disability that would be an important evidence reference to think the equity issue of the national preventive health service. On the priority of health care system setting, how to pursue equity of access to health care as far as possible is an importance issue [18, 34]. Inequity in access to preventive health service has been considered to be closely associated to differences in age, family, income, gender, race/ethnicity, urban/rural residence, severity of disability, and education level [29, 32, 38, 39]. In Taiwan, the government launched the National Health Insurance (NHI) program in 1995 to provide compulsory universal health care coverage including medical care service and preventive health services. Nowadays, the NHI enrolls over 99.9 % of Taiwanese population and has contracts with 93.68 % of all medical providers [33] (National Health Insurance Administration, 2015). Since the launch of the NHI, investigations have reported there to be obvious advance in terms of equity of access to health care, greater financial risk protection, and the geographical distribution of physicians [27, 28, 42]. However, the inequality of access to preventive health care still exist among people with disability, such as women with visual impairment and we try to explore the utilization and associated barriers which need to break through as following.

### The utilization of Pap smear for women with visual impairment

Our study discovered the rate of women with visual impairment ever received the Pap smear screening was 66.5 % and that in the previous 3 years is 44.3 % in Taiwan. Armour et al. [3] had survey by phone call for women with disability above 18 years old, there was 78.9 % ever using Pap smear in the previous 3 years that higher than the rate of women with visual impairment in the present study. According to the National Health Interview Survey of Taiwan, the overall prevalence of undertaking a Pap smear screening test is 69 % in 2009. This finding showed the utilization of Pap smear in women with visual impairment still lower than general population even providing free once Pap smear screen in 3 years to women over 30 years old by HPA [12] in Taiwan that because of lacking cognition and environmental barriers probably. In addition to the utilization of Pap smear, we also found the age of the first received Pap smear screening test was 38.8 years old with extremely older than 21 which is the recommended age to begin screening [35].

### The associated factors of using Pap smear among women with visual impairment

Many studies has indicated that the age [6, 15, 16, 33, 40] and educational level [4, 11, 21] are the associated factors of using Pap smear for general women population and women with disability. That the same as our results for women with visual impairment.

#### The age and educational level

Huang et al. [16] showed the peak received rate of Pap smear among women with disabilities is between age 40 and 49 (11.64 %). Our study found the peak rate of Pap smear screening test in women with visual impairment is located over age 55 to 64. In a cohort study conducted among 150,052 women aged 15 years or older, the highest incidence of cervical intraepithelial neoplasia (CIN) three occurred among those aged 25 to 29 years [17]. We should pay more attention on this topic to get the most benefit of this preventive medical service. Furthermore, the health policy maker should consider to provide HPV typing co-test according to the guideline in such lower utilization group those have multiple barriers in access to preventive medical service.

Some studies showed the lower the educational level of women with disabilities, the lower utilized rate of Pap smear screening test [6, 15, 16, 26, 40]. There are strong and consistent evidences to point out that educational attainment affects an individual’s health-related behaviors [4, 11, 21]. Evidence reported previously illustrated that health literacy, defined as an individual’s ability to obtain, process, and understand basic health information and services needed to make appropriate health decisions [36], maybe a more significant factors than educational attainment in explaining and predicting individual health behaviors and use of health service [8]. How to improve the health literacy is a good way to decrease the inequality among visually disabled women in utilization of Pap smear screening test.

#### Income

Some studies showed the usage rates and frequency of preventive health service was direct proportional to income [13, 33]. But our study showed income is not an influencing factor in utilization Pap screening test in women with visual impairment. This implies that the Bureau of Health Promotion provides the free Pap smear screening test decrease the inequality of utilization. It should be noted that the income would be discussed in some countries where Pap smear screening test is a non-national public health services. Lantz et al. [20] believed that income did not directly determine Pap smear screening test behaviors, but together with other factors exerted an indirect influence.

#### The other associated factors for women with visual impairment

We found that marital status, unemployment, severity of disability, and gynecologic experience were significantly associated factors.

Unmarried visually disabled women had lower Pap smear screening test utilized rates, which is similar with many previous studies [6, 13, 16, 20, 33]. It is likely that Taiwanese and Asian people take comparably conservative attitudes toward sex and knowledge of sexual organ compared to the public in Western countries, causing the unmarried disable women to be unwilling to receive this preventive health services. This result is also responded the barriers of Pap smear using among our samples.

Our study illustrates the severity of visual impairment is a significant factor as utilization of barrier in Pap smear screening test [14] indicated the utilization of preventive health service in people with visual impairment is also lower with the severity of disabilities (from 19.16 to 2 %) [14]. And the other survey found that the utilization of Pap smear screening test in women with disabilities also decreases with the severity of disabilities (from 10.19 to 5.47 %) [16]. So the severity of various disabilities would be considered by health policy makers.

According our logistic regression analysis result, we found the gynecologic experience was a significant factor of Pap smear using for women with visual impairment (OR = 4). This finding not only tells us the well-responsible gynecologist in Taiwan but also reveals the gynecology OPD is an important role in promotion of women’s reproduce health. Regarding to the reasons of non-utilization of Pap screening test in women age 30 or more, we found the common excuses is they thought it is unnecessary because of young age (22.3 %) and they felt nothing abnormal (12.6 %).

Recent evidences showed HPV infection is the most important risk factor for cervical cancer and scientists believe a woman must be infected by HPV, especially high-risk types, before she develops cervical cancer. Note worthily, HPV can be passed from one person to another during skin-to-skin contact.

This implies such group has the wrong recognition about cervical cancer and what we need focus on this area. The educational media should emphasize to correct these wrong recognitions and prevent women to ignore the Pap smear screening test due to such incorrect knowledge.

But it should be noted that the marriage status may correlated with “main care giver” or/and “Gynecology experiences” within our samples in the Table 3. So we excluded the marriage in the model even the auto-correlation via independent variables of logistic regression can be neglected. The finding was that the samples with Gynecology experiences, the minor disability and the main care givers are not mothers/sister were higher pap smear utilization and the Gynecology experiences was still the strongest factor to predict the Pap smear using for women with visual impairment in the present study.

### The cognitions, attitudes and barriers of Pap smear utilization among women with visual impairments

The other promoting factors are the attitudes of doctors and nurses which were more important than other factors in the present study. Barriers to receive Pap smear screening test for women with visual impairment can be addressed through interventions aimed at improving on cognitions and attitudes for cervical cancer risk factors like the association of age, sexual experience and cervical cancer.

From our findings in the present study, the Gynecology experiences of women with visual impairment is a key factor for Pap smear using, maybe the minor disability and the main caregivers would be potential factors. So how to promote their Pap smear using especially for them without Gynecology experiences is the most important issue. The health policy decision makers must be much effort on health education about Pap smear during school period which include the special education school. And to increase the support classes relative to Pap smear to their caregivers by other prevention health care or other medical utilization opportunities.

## Conclusion

The current study investigated the utilization and barriers of Pap smear screening test in women with visual impairment. Totally, 66.5 % of participators were ever using Pap smear in this study and 38.9 % used it during pass 1 year that certainty lower than general population. The first time to accept Pap smear was 38.8 year old among our participators with using Pap smear that is extremely older than the recommended age to begin screening. The gynecological experience was a key factor for women with visual disability in Taiwan so that would be as a promoting factor to them.

By providing an understanding of the factors influencing women with visual impairment to take the Pap smear screening test, our study also serves as a reference resource for erasing the barriers and inequality among the visually disabled women.
